# A Method to Distinguish Chromium‐Tanned Leathers With Low and High Risks of Surface Hexavalent Chromium

**DOI:** 10.1111/cod.14729

**Published:** 2024-12-03

**Authors:** Ivan Chen, Jonas F. Hedberg, Yolanda S. Hedberg

**Affiliations:** ^1^ Department of Chemistry The University of Western Ontario London Ontario Canada; ^2^ Surface Science Western, the University of Western Ontario London Ontario Canada

**Keywords:** allergic chromium dermatitis, hexavalent chromium, humidity, leather, reduction, speciation, vegetable tannins

## Abstract

**Introduction:**

Traces of hexavalent chromium, Cr(VI), are a major concern for skin contact with Cr‐tanned leather. Current extraction methods (ISO 17075‐1:2017) for Cr(VI) testing do not necessarily reflect the true potential of surface‐formed Cr(VI), as extracted concentrations are dependent on previous storage and atmospheric conditions.

**Objectives:**

To test whether a spiking method protocol can distinguish leathers with high and low risks of releasing Cr(VI).

**Methods:**

Two groups of leather types were selected based on previously detected Cr(VI) (group A) and optimal tanning practices with high antioxidants (group B), corresponding to a high and low risk of forming and keeping Cr(VI). Leathers were spiked with different concentrations up to 10 mg/kg of Cr(VI) and incubated at 80°C for 24 h prior to the ISO 17075‐1:2017 extraction protocol.

**Results:**

All Cr(VI) was reduced by group B leathers, whereas all group A leather extracts contained detectable Cr(VI) that was dependent on the exact leather type and the amount initially spiked.

**Conclusion:**

Pre‐treatment of samples with supplemental Cr(VI) is a potential method for determining the reduction capabilities of leather, which are closely related to the risk of Cr(VI) formation. 10 mg/kg spiking unambiguously distinguished leathers with high and low risks of forming Cr(VI).

## Graphical abstract

Environmental factors influence standard testing of hexavalent chromium in leathers, which leaves an uncertainty of whether a leather might form hexavalent chromium during use. We present a method to distinguish leathers with high and low risks of forming hexavalent chromium on their surface.

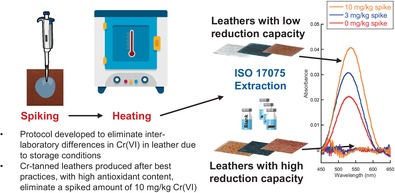

## 1 Introduction

Chromium (Cr) salt can be used as a mineral tanning method for leather and is a major tanning method worldwide [1]. While leather is tanned with a trivalent Cr salt, Cr(III), more specifically basic Cr(III) sulfate (Cr(OH)SO_4_), traces of hexavalent Cr, Cr(VI), can be formed under some circumstances [2]. Cr(VI) is of primary concern from a human health perspective, as it has high skin diffusion rates, is carcinogenic, and is a strong sensitizer [3, 4, 5, 6, 7, 8]. Most national and international regulations of relevance to leather products currently target Cr(VI), not total Cr or Cr(III). In North America, Proposition 65 in California prohibits Cr(VI) for products that can potentially cause skin exposure if they do not clearly warn consumers [9]. Recently (in 2021) [10], the European Chemicals Agency (ECHA) lowered the limit of Cr(VI) in leather from 3 to 1 mg/kg, to be mandated by REACH regulations within 5 years. These are low values compared to the amounts of total Cr and Cr(III) in leather [2]. Yet in certain environments, for example, alkaline environments, dry air, or under UV irradiation, some of this Cr(III) is oxidised to Cr(VI) [11, 12]. This Cr(VI) might be detected by an extraction method, such as ISO 17075‐1:2017 [13], if the leather reduction capacity is low. The leather reduction capacity is here defined as a measure of the leather's ability to reduce any formed Cr(VI) back to Cr(III). It can be compared to the reduction capacity of solutions and solids in solution [14, 15]. A higher reduction capacity of the leather lowers the probability of the formation and stability of Cr(VI) [16, 17, 18], and hence skin exposure to Cr(VI).

The chemical form and amount of Cr(VI) formed on the leather surface are highly dynamic and influenced by factors such as relative humidity and temperature [11, 16, 17, 19]. Therefore, even the same leather might result in different laboratory results when testing for Cr(VI), unless there are measures taken to exactly control the storage and pre‐conditioning environmental variables. Leathers free of antioxidants in dry conditions (< 35% relative humidity) are particularly susceptible to the formation of Cr(VI) [11, 19]. In practical settings, leathers are exposed to varying humidity, UV irradiation, and temperature conditions in ways not captured by the predominant testing protocol ISO 17075‐1:2017 [13]. Cr(VI) has been found to have reformed on leather samples with low reduction capacities, because of low antioxidant contents, after up to 12 months of storage at room temperature [17] or for up to 8 months under simulated use conditions [12]. This means that some leather products that showed non‐detectable Cr(VI) levels during testing later can form and release Cr(VI). A method that accurately assesses the reduction capacity of leathers might hence provide directly relevant information for consumers and circumvent the limitations posed by the variability in laboratory testing.

It is well‐known that the tanning protocol affects the risk of Cr(VI) detection and formation [20]. The pre‐treatment of leathers with additions of fatty acids, vegetable tannins, and reducing agents, can dramatically impact the leathers' reduction capacity, as summarised in a previous review [2]. This fact is considered in different international associations' ratings, such as the gold rating of the Leather Working Group [21].

The present study proposes a new method using a spiking and standardised pre‐conditioning method prior to ISO 17075‐1:2017 testing to distinguish leathers with a high and low risk of formation of Cr(VI).

## 2 Methods

### 2.1 Cleaning Procedures and Chemicals

All glassware was acid‐washed using 10% nitric acid for 24 h, followed by four rinses with ultrapure water (resistivity of 18.2 MΩ cm). Phosphoric acid (99.99%) was obtained from Sigma Aldrich, Canada. A 10 mg/L Cr(VI) standard in water was obtained from Delta Scientific Laboratory Products, Canada. Potassium hydrogen phosphate trihydrate (99%) was obtained from Fisher Scientific, Canada. All chemicals were of analytical grade purity.

### 2.2 Leather Specifications

Two groups of leathers that have previously been tested in our laboratory were selected based on their variance in tanning practices. Leathers denoted as ‘A’ represent a group of leathers with limited reduction capacity with respect to Cr(VI), as samples from these leathers released detectable Cr(VI) at least once previously in our laboratory. A1 was investigated previously, summarised under the name ‘L1’ in a review referring to other studies of this leather [2]. Leathers tanned with best practices or known high antioxidant content, denoted ‘B’, represent a group of leathers that had vegetable tannins or antioxidants added in the post‐tanning process and leathers that were produced by tanneries certified as Gold by the Leather Working Group (B2–B3) [21]. B2–B3 were obtained from California and sent to our laboratory from the Centre of Environmental Health as tannery samples produced with the Gold rating of the Leather Working Group. B1 has previously been proven to have a high reduction capacity for Cr(VI) and its previous studies were reviewed and summarised under the name ‘L3’ [2]. Table 1 lists the leathers used in this study, including the sample source and relevant information on existing legislation, although most of the tanning details are unknown. The experimenters were blinded, which means the leather sample categories were unknown to the researcher during the testing.

**TABLE 1:** General information of the 10 leather samples assessed.

Leathers	Sample details (all samples were unused and new)
A1	Unfinished leather intended for work gloves. Tanning included no vegetable tannins or antioxidants. Tannery sample produced in Europe, before 2015 (REACH not in force).
A2	Retrieved from work gloves. Sold in California after Proposition 65 came into force.
A3	Retrieved from sandals. Sold in California after Proposition 65 came into force.
A4	Retrieved from shoes. Sold in California after Proposition 65 came into force.
A5	Retrieved from shoes. Sold in California after Proposition 65 came into force.
A6	Retrieved from shoes. Sold in California after Proposition 65 came into force.
A7	Retrieved from work gloves. Sold in California after Proposition 65 came into force.
B1	Tanned with vegetable post‐tannins. Tannery sample produced in Europe before 2015.
B2	Treated with antioxidants in the post‐tanning step, also contains vegetable tannings. Produced according to Leather Working Group Gold state‐of‐the‐art protocols. Obtained from California as tannery leather sample after Proposition 65 came into force.
B3	Treated with antioxidants in the post‐tanning step, also contains vegetable tannings. Produced according to Leather Working Group Gold state‐of‐the‐art protocols. Obtained from California as tannery leather sample after Proposition 65 came into force.

*Note*: Group A: Included because of previously detectable Cr(VI) of samples of this leather type and European or Californian origin. Group B: Included because of Leather Working Group gold rating (B2–B3) or previously confirmed high antioxidant content (B1).

### 2.3 Leather Exposure Conditions

**FIGURE 1:**
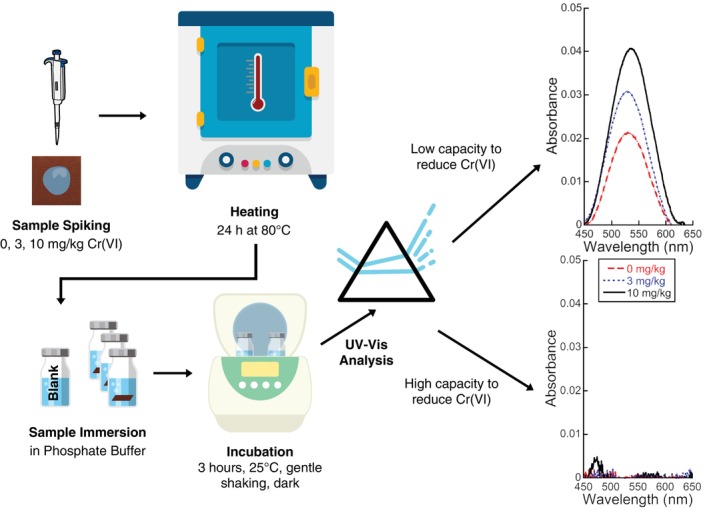
Schematic illustration of the experimental protocol. Adapted from the ISO 17075‐1:2017 method for colorimetric determination of chromium in leathers. Modifications are visually represented (spiking and heating). Curve fitting is visually depicted through two examples of UV–vis spectra (to the right) produced by A3 (top) and B1 (bottom) leather samples for non‐spiked (0 mg/kg), 3 mg/kg, and 10 mg/kg Cr(VI) pre‐treatments.

Modifications were applied to the ISO 17075‐1:2017 Cr(VI) extraction protocol to investigate methods of leather reduction capacity testing, as follows and depicted in Figure 1. A drop of Cr(VI) was applied on the rough side (flesh side) of the leather that is meant to be in contact with, or closest to, the skin. That flesh side also absorbs water droplets more easily, facilitating the spiking procedure. The surface area was kept constant at 2.4 cm^2^ (one side of 1 cm^2^), as it is the surface area (and not the mass) that determines the amount of surface‐formed Cr(VI) [11, 22]. The dry weight differed between 0.03 and 0.11 g and was measured for each piece and accounted for. All leathers were placed into dry glass vials and triplicates of each leather type were treated with either 0, 3, or 10 mg/kg Cr(VI) using corresponding droplet volumes with Cr(VI) stock (10 μg/mL) based on weighed mass of each dry leather sample. Larger volume droplets were applied as two smaller droplets with the first droplet not exceeding 50 μL. The second droplet was applied after evaporation of the first in the oven. All samples were placed into the oven and left uncapped for 24 h at 80°C. The high temperature guarantees a low relative humidity (< 10%), and this treatment is a known and recommended worst‐case pre‐treatment for Cr(VI) analysis of leather, following ISO 10195 [23]. Immediately after the treatment period, the vials were cooled to room temperature for 30 min and submerged with the phosphate buffer (PB) for extraction. PB served as the extraction buffer, composed of 22.8 g/L K_2_HPO_4_∙3H_2_O. The pH was 8 ± 0.1 adjusted by phosphoric acid and de‐aerated with N_2_ gas for 20 min. Extraction was performed in capped glass vials with leathers completely submerged in 5 mL of PB incubated for 3 h in a rocking incubator (VWR) at 25°C with an 11°tilt and 25 cycles per minute. The PB was collected after incubation and analysed directly or stored at −20°C until the samples were analysed with UV–Vis spectroscopy.

### 2.4 UV–vis Spectroscopy

Calibration curves for UV–Vis spectroscopy were generated using a 10 μg/mL Cr(VI) standard to obtain the following 10 mL standard concentrations: 60, 90, 125, 250, 500, 1000 μg/L. A Sartorius analytical balance was used to record the weight of the added Cr(VI) and the final volume. The samples were analysed by UV–Vis spectrometer (Cary 60, Agilent) immediately after preparation and calibration. All calibration curves were linear (*R* ^2^ > 0.999).

Determination of Cr(VI) concentrations involved a pink complex formed with diphenylcarbazide (DPC) and phosphoric acid with volume ratios corresponding to 96 vol.% sample, 2 vol.% DPC, and 2 vol.% phosphoric acid. A DPC solution was prepared in accordance with ISO 17075‐1:2017. 1 g of 1,5‐diphenylcarbazide (Sigma Aldrich, Canada) was dissolved in 10 mL of acetone 99.9% (Fisher Scientific, USA) and acidified with 5 μL of 99.99% glacial acetic acid (Sigma Aldrich, Canada). The solutions were made freshly and used for up to 2 weeks when stored in opaque glass vials at 4°C.

Samples were left for 10 min with periodic agitation. Upon formation of pink complexes, the UV–visible spectrometer analysed them for Cr(VI) concentration at 540 nm. Based on the noise levels of the UV–Vis spectra, the limit of detection (LOD) was estimated to be 1 mg/kg (Cr(VI)/leather). The LODs were qualitatively verified during analysis of raw spectroscopic data, as values above the LOD produced a clear and distinct peak at 540 nm.

Raw spectroscopic data for each sample was plotted in Origin (2016). At the base of the beginning and end of the peaks, a straight line was drawn between the two points which served as the baseline. The baseline was subtracted from peak height (absorbance) at 540 nm. The peak height was converted to Cr(VI) concentration (mg/L) using the corresponding linear calibration curve constructed by the Cr(VI) standards. All blank (background control) samples (with no leather piece) revealed no detectable concentrations and were therefore not subtracted from the sample concentrations. Finally, the Cr(VI) concentration was multiplied by the solution volume and divided by the dry leather weight in order to obtain the final results as mg Cr(VI)/kg dry leather. The average and standard deviation of three independent samples were then calculated and presented in the following.

### 2.5 Statistical Analysis and Data Presentation

Student's *t*‐tests of unpaired data with unequal variance were run between two sets of triplicate leather samples. Significant differences are denoted as significant when *p* < 0.05.

## 3 Results

**FIGURE 2:**
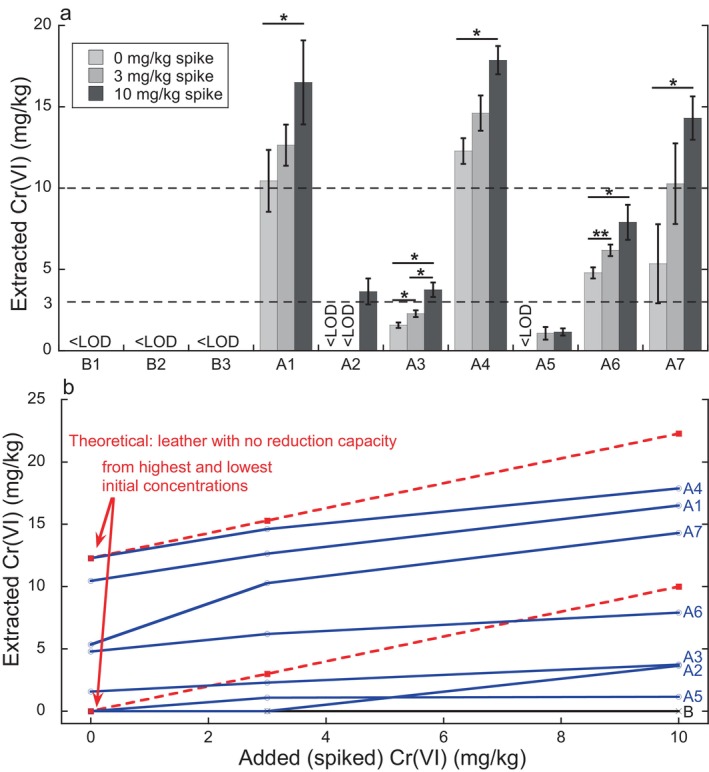
(a) Extracted Cr(VI) from non‐spiked (0 mg/kg), 3 mg/kg spiked, and 10 mg/kg spiked samples across 10 leather types. < LOD represents Cr(VI) concentrations below the limit of detection. The error bars represent the standard deviation of independent triplicate samples. Statistically significant differences between spiking concentrations (*p* < 0.05) are denoted with an asterisk (*). The two dashed lines correspond to 3 and 10 mg/kg extracted Cr(VI). (b) Extracted Cr(VI) as a function of added Cr(VI) together with curves (red dashed lines) representing theoretical samples without any reduction capacity for Cr(VI). Corresponding raw data and values in μg/cm^2^ are given in Table S1.

Figure 2 shows the quantified, extracted values of Cr(VI) for the 10 different leather types spiked with 0, 3, and 10 mg/kg of Cr(VI). There was no detectable Cr(VI) recovered for the leathers in the ‘B’ category. This means that these leathers reduced all added (spiked) Cr(VI) completely, showing a very high reduction capacity, Figure 2. In contrast, Cr(VI) was detected for the highest spiking concentration (10 mg/kg) for all ‘A’ leathers. Among the 7 different A leathers, four released high (> 3 mg/kg) Cr(VI) amounts without any addition of Cr(VI). The other three A leathers released Cr(VI) levels at 1.6 mg/kg and undetectable Cr(VI) levels without any addition of the Cr(VI) (no spiking), Figure 2. For all seven A leathers, the highest concentration was detected for the highest spiked (10 mg/kg) amount of Cr(VI) and there were increasing amounts of Cr(VI) extracted for increasing spiked amounts. These changes were statistically significant for all leathers except those that had no detectable Cr(VI) amounts for their non‐spiked leather pieces, Figure 2a. However, the increase of extracted Cr(VI) with increasing spiked Cr(VI) (Figure 2b) was not the same for the different A leathers and no leather recovered all the Cr(VI) that was spiked (the difference between the 10 and the 0 mg/kg spiked samples was less than 10 mg/kg for all leathers investigated). This means that all A and B leathers had a lower slope in Figure 2b compared with theoretical samples that have no reduction capacity for Cr(VI). The leather with the least ability to reduce Cr(VI) (A7), which means the highest average slope in Figure 2b, still reduced 11% of it, while the optimally tanned leathers (group B) reduced 100% of the spiked Cr(VI), with the lowest slope in Figure 2b.

Extracted Cr(VI) levels without any spiking were 12.3, 10.45, 5.4, and 4.8 mg/kg for A4, A1, A7, and A6, respectively. These values correspond to 0.16, 0.19, 0.12, and 0.09 μg/cm^2^ Cr(VI), Table S1. The leathers that released high amounts of Cr(VI) formed on their surface during the pre‐conditioning at dry air in the oven also showed a high slope in Figure 2b, corresponding to a reduction capacity of less than 70% of a total spiked amount of 10 mg/kg Cr(VI). Vice versa, a high ability to reduce Cr(VI) (complete reduction of 10 mg/kg spiked Cr(VI)) correlated in all cases with undetectable values of Cr(VI).

## 4 Discussion

This spiking and pre‐conditioning method proposed here clearly distinguished leathers that have a low and a high risk of the formation of Cr(VI). This avoids false negative results. For example, the leather A1, which showed one of the lowest reduction capacities and one of the highest intrinsic (formed during the oven aging procedure) Cr(VI) values has been tested by the ISO 17075‐1:2017 protocol for different pre‐conditions previously, resulting in undetectable (in most cases) up to 25 mg/kg Cr(VI) values, depending on the pre‐conditions [11]. It was enough to keep it for 24 h in air with a relative humidity greater than 35% to obtain undetectable values [11]. A typical laboratory environment has a relative humidity greater than 35%, which often results in false negative results for leathers that might later be able to form Cr(VI) on their surface once they are in warmer and/or drier environments. Relative humidity can be decreased by several methods, including desiccators, climate chambers, and increasing the temperature. The latter is the only way that is accessible in most labs and standardised, through ISO 10195. Our previous studies on the A1 leather showed that it was the relative humidity, not the temperature, that mattered most and that there was no significant difference between pre‐conditioning at 70°C or 25°C if the relative humidity was kept constant (at 20%) [11]. Therefore, we recommend the use of ISO 10195 (24 h dry exposure at 80°C) prior to any Cr(VI) extraction from leathers by the ISO 17075 test method.

Literature findings agree with a greater reduction capacity for leathers that have been vegetable‐tanned or treated with antioxidants, and with a correlation between a higher reduction capacity of the leathers and a lower risk of the formation of Cr(VI) [16, 17, 18, 19, 20, 24, 25, 26, 27, 28, 29, 30, 31, 32, 33, 34, 35, 36]. Though ISO 17075 is suitable for testing the presence of Cr(VI), the protocol cannot assess a leather sample's capacity for reducing Cr(VI). Since laboratory conditions may not reflect environmental conditions that are important for the formation of Cr(VI), measuring the reduction capacity of leathers is important.

Cr(VI) on the surface of Cr‐tanned leathers forms from the large pool of available Cr(III), which is several mass percentages of Cr‐tanned leather. In this study, this air‐formed Cr(VI) ranged up to 0.19 μg/cm^2^. Assuming that this leather is directly touching the skin for 48 h, this level of Cr(VI) would be enough to elicit allergic contact dermatitis to Cr in more than 50% of Cr‐allergic persons, according to a reported minimum elicitation threshold (50%) value of 0.15 μg/cm^2^ [37]. Likewise, the lowest detected air‐formed (non‐spiked) Cr(VI) value for group A leathers was 0.06 μg/cm^2^ for A3, which would be enough to elicit a positive reaction in more than 10% Cr‐allergic persons according to a previously reported value of 0.03 μg/cm^2^ [37].

A fundamental difference between European [10] and Californian [9] chemical legislation is whether the consumer needs to be informed about the chemical risks. The relevant legislation in the European Union specifies a restriction limit (recently lowered to 1 mg/kg), while Californian legislation allows any values as long as the consumer is warned. In any case, accurate testing is necessary, especially since even the now suggested 1 mg/kg Cr(VI) restriction limit corresponds for typical leather thicknesses and densities to 0.01–0.05 μg/cm^2^, which is close to the 10% minimum elicitation limit [37] of 0.03 μg/cm^2^.

The proposed method cannot identify the specifics of the antioxidants/tannins nor determine how long the leather's reduction capacity would last. Studies have shown that the reduction capacity can decrease or change with time [12, 17, 38], although it is unknown if those studies were done on leathers that would be equivalent to leathers produced in a state‐of‐the‐art Gold Leather Working Group rated tannery. To test long‐term exposure scenarios, a long‐term sequential protocol and a protocol simulating the washout of antioxidants [12, 17, 38] can be used. While spiking methods are commonly used to validate analytical methods or to measure the reduction capacity of solutions or soils, this protocol is the first to use it for consumer products. The concept could, in the future, be used for other consumer products, which are suspected to change the bioavailability and chemical form of potentially sensitising substances.

## 5 Conclusion

The aim of this study was to test whether spiking of leather samples could be used to differentiate leathers of high risk and low risk for Cr(VI) formation. The following main conclusions were made:

1. The tested leathers treated with antioxidants or vegetable tannins, and produced by Leather Working Group Gold certified tanneries, yielded no detectable levels of Cr(VI) and reduced spiked Cr(VI) (up to 10 mg/kg) to levels not detectable at a limit of detection of 1 mg/kg.
2. Leathers that previously released detectable levels of Cr(VI) at least once in our laboratory could not fully reduce the spiked 10 mg/kg Cr(VI). They reduced it by 11%–88%.
3. The proposed aging and spiking protocol allows for distinguishing leathers with high and low risks of the formation of Cr(VI).

## Author Contributions

**Ivan Chen:** investigation, writing – original draft, visualization, writing – review and editing. **Jonas F. Hedberg:** conceptualization, investigation, methodology, validation, visualization, writing – review and editing, supervision. **Yolanda S. Hedberg:** conceptualization, funding acquisition, writing – original draft, methodology, validation, visualization, writing – review and editing, project administration, supervision.

## Ethics Statement

There are no ethical considerations associated with this study.

## Conflicts of Interest

The authors declare no conflicts of interest.

## Supporting information


Data S1.

## Data Availability

All data generated or analysed during this study are included in this published article and its Supporting Information.
