# Supplementary material for: A Method to Distinguish Chromium‐Tanned Leathers With Low and High Risks of Surface Hexavalent Chromium
Source: Contact Dermatitis. 2024 Dec 3;92(3):234–40. doi: 10.1111/cod.14729 (PMC11795338; doi:10.1111/cod.14729)
Supplement: Supplementary file 1 — Data S1. [file COD-92-234-s001.docx]

**Supplemental file**

**A Method to Distinguish Chromium-Tanned Leathers with Low and High Risks of Surface Hexavalent Chromium**

Ivan Chen,^1^ Jonas F. Hedberg,^2^ Yolanda S. Hedberg^1,2,^*

^1^Deptartment of Chemistry, The University of Western Ontario, 1151 Richmond Str., London, Ontario,
N6A 5B7, Canada

^2^Surface Science Western, The University of Western Ontario, 999 Collip Circle, London, Ontario,
N6G 0J3, Canada

**Table S1**. Average and standard deviations are shown for triplicate samples for each leather type for non-spiked, 3 and 10 mg/kg spiked leather samples in two different units, normalized to the dry leather weight and the surface area of the leather.

|  | Non-spiked | | Spiked with 3 mg/kg Cr(VI) | | Spiked with 10 mg/kg Cr(VI) | |
| --- | --- | --- | --- | --- | --- | --- |
| Leather | mg/kg Cr(VI) | µg/cm² Cr(VI) | mg/kg Cr(VI) | µg/cm² Cr(VI) | mg/kg Cr(VI) | µg/cm² Cr(VI) |
| A1 | 10.5±1.9 | 0.188±0.020 | 12.6±1.3 | 0.24±0.012 | 16.5±2.6 | 0.31±0.013 |
| A2 | <LOD | <LOD | <LOD | <LOD | 3.7±0.80 | 0.051±0.022 |
| A3 | 1.6±0.17 | 0.063±0.006 | 2.3±0.21 | 0.105±0.010 | 3.8±0.44 | 0.141±0.012 |
| A4 | 12.3±0.79 | 0.16±0.010 | 14.6±1.1 | 0.17±0.013 | 17.9±0.87 | 0.24±0.012 |
| A5 | <LOD | <LOD | 1.1±0.39 | 0.016±0.006 | 1.2±0.22 | 0.018±0.003 |
| A6 | 4.8±0.34 | 0.088±0.013 | 6.2±0.35 | 0.114±0.002 | 7.9±1.1 | 0.136±0.018 |
| A7 | 5.4±2.4 | 0.123±0.039 | 10.3±2.5 | 0.14±0.018 | 14.3±1.3 | 0.276±0.008 |
| B1 | <LOD | <LOD | <LOD | <LOD | <LOD | <LOD |
| B2 | <LOD | <LOD | <LOD | <LOD | <LOD | <LOD |
| B3 | <LOD | <LOD | <LOD | <LOD | <LOD | <LOD |
